# Percutaneous First Metatarsophalangeal Joint Fusion

**DOI:** 10.2174/1874325001711010724

**Published:** 2017-07-31

**Authors:** Thomas Bauer

**Affiliations:** Department of Orthopedic Surgery, Ambroise Paré University Hospital, West Paris University, 9 avenue Charles de Gaulle, 92100 Boulogne Billancourt, France

**Keywords:** Hallux rigidus, Percutaneous surgery, Metatarsophalangeal fusion

## Abstract

The first metatarsophalangeal (MTP1) joint fusion is a very useful procedure in forefoot surgery and is still the gold standard for the treatment of severe and painful hallux rigidus. Normal walking and running are possible after MTP1 fusion, the first ray mobility being essentially in the interphalangeal (IP) joint with a compensatory hypermobility in dorsal flexion. Percutaneous MTP1 fusion is a simple procedure providing comparable results to fusions performed with open techniques. Postoperative cares are simplified with an immediate full weight bearing on rigid flat shoes and quick return to normal walking. Bone preparation is an important step and requires an experience in percutaneous forefoot surgery. Arthrodesis positioning and fixation with this percutaneous procedure are simple with possibility of clinical and radiological control. The indications for percutaneous MTP1 fusion are very large and only severe bone loss or osteoporosis represent the limits for this technique.

## INTRODUCTION

The first metatarsophalangeal (MTP1) joint fusion is a very useful procedure in forefoot surgery and is still the gold standard for the treatment of severe and painful hallux rigidus. Normal walking and running are possible after MTP1 fusion, the first ray mobility being essentially in the interphalangeal (IP) joint with a compensatory hypermobility in dorsal flexion. [Mann [1], DeFrino [2]. The main difficulty in this procedure is the 3D positioning of the arthrodesis that should be adapted to global foot anatomy, daily activity and shoe wearing habits of each case [Conti [3], Harper [4], Alexander [5], Kelikian [6], Womack [7]. Another non specific difficulty is linked to the primary stability of the fusion depending on both technique for fusion, site preparation and type of internal fixation [Kelikian [6], Womack [7], Chana [8], Wu [9], Curtis [10], Rongstad [11], Watson [12], Goucher [13]. Several open or arthroscopically assisted procedures for MTP1 arthrodesis have been described with fusion rates from 90% to 100%. Recently, percutaneous MTP1 fusion techniques were described with good results and less morbidity [Bauer [14-17], Fanous [18]. The authors present the technique and results of a percutaneous MTP1 fusion and discuss the benefits and indications.

## OPERATIVE TECHNIQUE

### Instruments

Surgical tools for percutaneous MTP1 fusion are identical to those used for all percutaneous forefoot surgical procedures including a conic burr, a Beaver^®^ blade, elevators, rasps, low speed and high torque drill and a fluoroscope. For the fixation cannulated 3.0 mm compression screws were used in the described technique but other percutaneous fixation systems can be used.

### Patient Set Up

The patient is in supine position, under regional or local anaesthesia, with the foot free over the end of the table to allow AP and lateral fluoroscopic control.

### Portals

**Fig. (1):**
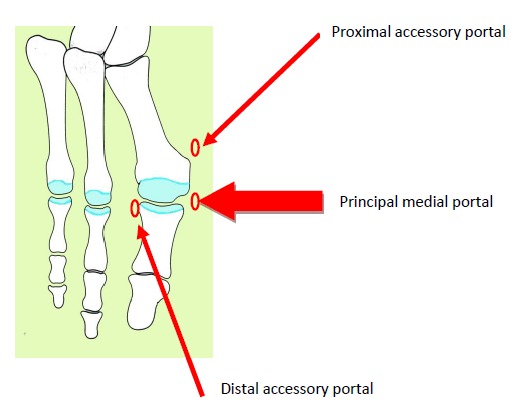
Portals.

Percutaneous MTP1 fusion is performed with one main portal and 2 accessory portals (Fig. **1**). The main portal is medial at the MTP1 joint line level and is used for the preparation of bony areas. Two accessory portals can be useful in some cases:

one proximal medial and plantar portal at the level of the first metatarsal head can be used for dorsal and medial osteophytes removal and one distal lateral and dorsal portal at the level of the first phalanx (P1) basis that can be used for dorsal and lateral osteophytes removal and for lateral MTP1 joint capsule and ligaments release.

### Method of Fusion Site Preparing

 The bony areas preparation begins with the removal of metatarsal or phalangeal osteophytes if necessary. This resection is performed through the main portal (sometimes for large dorsolateral osteophytes accessory portals are required) with the large conic burr after periosteal peeling off with the elevators to create a working area and avoid soft tissue damages. Bone debris is carefully evacuated with rasps and the resection site is abundantly cleaned with normal saline. The quantity and quality of osteophytes removal must be assessed under fluoroscopic control; this resection must be adapted to patient’s symptoms (dorsal and medial osteophytes often create impingement with shoes but lateral osteophytes are rarely symptomatic). An excessive resection with the risk of bone loss (most often on the first metatarsal head) must be avoided otherwise the primary stability of the arthrodesis would be compromised.

**Fig. (2):**
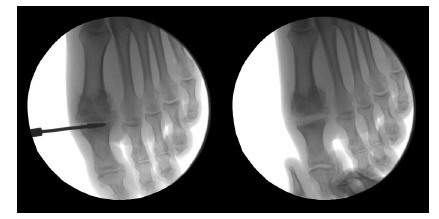
Bone surfaces preparation.

The preparation of the fusion site is one of the most important steps of this procedure and is performed through the principal medial portal (Fig. **2**). The conic burr is placed in the MTP1 joint with traction on the hallux. Cartilage resection and bony areas preparation are performed with the burr under fluoroscopic control to assess both quantity and quality of bone resection. In this technique, the fusion site is prepared by cutting two flat and parallel surfaces. The preparation of this area is the most difficult step of this procedure and the main risk is to have an asymmetrical resection. Some pitfalls must be avoided:

Excessive metatarsal bone resection: the bone of the proximal phalanx is more dense than the bone of the metatarsal head and the burr will tend to remove the weakest bone, on the metatarsal side. The risk is to obtain an excessive bone resection on the metatarsal head with first metatarsal shortening, loss of primary stability, metatarsus elevatus positioning of the arthrodesis with an increased risk of transfer metatarsalgias. It is thus important to control the burr and press more on the proximal phalanx than on the metatarsal head and assess the progression of the resection with fluoroscopic control.

Excessive dorsal resection: it is often more difficult to reach the plantar part of the MTP1 joint than the dorsal part and the burr will tend to remove the easiest to reach bone, on the dorsal part of the joint. The risk is to obtain an asymmetrical V-shaped bone resection due to an excessive dorsal resection with loss of primary stability and positioning of the arthrodesis with excessive dorsal flexion of P1. Bone preparation with the burr must be performed with a continuous traction on the hallux to open the MTP1 joint, to facilitate the access on the plantar part, to control bone resection and have parallel cuts on lateral fluoroscopic view.

After bone resection, the bone debris is evacuated with rasps and the arthrodesis site is abundantly washed with normal saline to avoid prolonged inflammation.

### MTP1 Arthrodesis Positioning

**Fig. (3):**
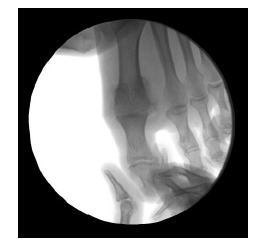
Arthrodesis positioning on AP view.

Contact between P1 and M1 is obtained by pressure in the axis of the first ray and the position is maintained with an oblique K-wire. The positioning of the arthrodesis is assessed clinically and under fluoroscopic control:

On AP view (Fig. **3**): first ray alignment or slight valgus, first ray length, metatarsophalangeal bone contact, no subluxation.

**Fig. (4):**
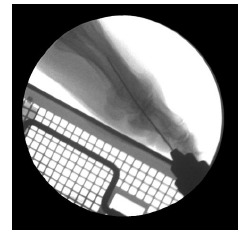
Arthrodesis positioning on lateral view.

On lateral view (Fig. **4**): P1 position is assessed with reference to the floor plane materialized by a metallic support applied on the sole of the foot. P1 must be parallel to the floor plane with a good bone contact and no plantar subluxation.

### Arthrodesis Fixation

**Fig. (5):**
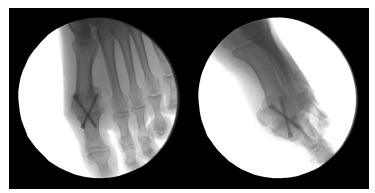
Arthrodesis fixation.

**Fig. (6):**
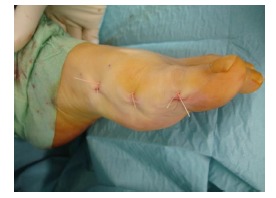
Postoperative view after closure.

 Percutaneous MTP1 fusion is fixed with 2 cannulated compression screws (Fig. **5**). The first K-wire is oblique from P1 to M1 (from medial-distal to lateral-proximal) and the second is oblique from M1 to P1 and crosses the first K-wire at the level of the first metatarsal head. The 2 cannulated screws are inserted and compression is obtained alternately on each screw. The stability of the MTP1 arthrodesis in dorsal and plantar flexion is then controlled and all the portals are closed (Fig. **6**).

### POSTOPERATIVE CARE

Percutaneous MTP1 fusion is performed in outpatients. The first dressing is changed after 10 days and then a simple dressing is applied with a cohesive bandage. Immediate full weight bearing is authorized with a postoperative shoe (with a complete flat and rigid insole). X-ray controls are done after 10 days and 1 month. Normal shoe wearing is begun after 1 month according to the clinical and radiological control. Sports activities are authorized after 1 month.

## INDICATIONS - RESULTS

The indications for percutaneous MTP1 fusion are basically the same as for open MTP1 fusion. This procedure is mainly performed for the treatment of severe and painful hallux rigidus and functional improvement is better and faster achieved in case of painful and stiff hallux rigidus with a compensatory hypermobility of the IP joint. Without a preoperative IP joint hypermobility return to normal walking and shoe wearing can be longer and sometimes painful due to the progressive adaptation of the IP joint (with inflammation and pain on the IP joint). Percutaneous MTP1 fusion can be performed for severe hallux valgus deformity, symptomatic hallux varus, complex forefoot deformities (in case of rheumatoid arthritis) or for failed previous forefoot surgery. The main limit for a percutaneous MTP1 fusion is the presence of an extensive bone loss with a short first ray and indication for a bone graft.

**Fig. (7):**
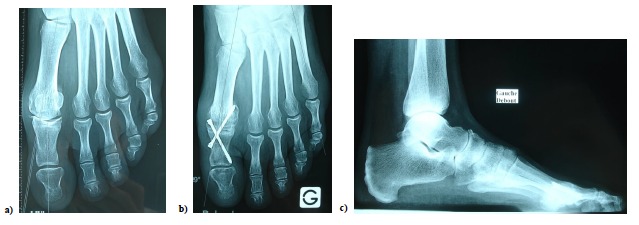
a) hallux rigidus. b) postoperative radiological control (2 months) c) lateral view (2 months).

Thirty two percutaneous MTP1 joint fusions were first analyzed in a preliminary prospective continuous series including 30 patients of an average 66 years old [Bauer (14)]. The indications for MTP1 joint fusion were symptomatic hallux rigidus or hallux rigido-valgus in most of the cases. All the patients underwent the same percutaneous procedure, in one-day surgery for 26 cases. Clinical results were assessed with the functional AOFAS forefoot scoring system preoperatively and at the latest follow-up. Radiographical analysis was focused on the positioning and quality of bone fusion of the procedure. No patient was lost to follow-up and the mean follow-up was 18 months. The functional AOFAS score improved in all the cases from a mean 36/100 preoperatively to a mean 80/100 postoperatively (p = 0.02). Thirty cases were satisfied or very satisfied with the final outcome of the procedure, one patient was disappointed and one was not satisfied. For the satisfied or very satisfied patients, normal shoe wearing was achieved after a mean 50 days. The radiological fusion was obtained in 31 cases on 32. The postoperative mean dorsal flexion of the MTP1 joint fusion was 21° (min: 15°, max: 35°) (Fig. **7**).

We report our experience with this procedure as from 2005 up to now all the MTP1 fusions were performed with the above described percutaneous technique. More than 200 percutaneous MTP1 fusions were performed with the same protocol. No major morbidity linked to the portals was noticed. Two cases with infection at the site of fusion were managed nonoperatively with antibiotic alone and went eventually fine. Removal of the screws was necessary in almost 15% of the cases. Eight cases of painful non unions required new surgery and an iterative percutaneous MTP1 fusion was performed in all the 8 cases leading to a pain free fusion in 6 cases. Asymptomatic non union (pain free hallux, good functional result with persisting MTP1 joint line without screw breakage on radiological control after 1 year or more) occurred in an average 10% of the cases without requiring further surgery. Ten cases of painful fused MTP1 fusions occurred and were mainly due to a malposition of the hallux (excessive dorsal or plantar flexion). Percutaneous MTP1 re-arthrodesis was performed with the same technique and resulted in 6 good results on 10.

With this experience, 2 main groups of indications with different prognosis can be drawn:

The best results with the quickest recovery were obtained for patients with stiff and painful hallux rigidus with hypermobile IP joint, for hallux varus cases, for failed previous surgery and for severe deformities due to inflammatory arthritis (rheumatoid arthritis…). In these indications, patients were very satisfied with a pain-free hallux within 2 months, normal shoe wearing and return to sports activities were achieved during the 2^nd^ or 3^rd^ month. Patients were able to wear high heel shoes (5 to 7 cm). Fusion was achieved before 3 months with a fusion rate superior to 90%.

Mild to moderate results with longest recovery and high rate of complications were obtained for patients with severe hallux valgus deformity and for elderly patients. In these indications, there was a high rate of non union or fibrotic union (20%), with recurrence of the deformity and hardware displacement resulting in a fair functional result with shoe wearing limited to flat and large shoes and sometimes insoles and orthoses.

## DISCUSSION

The percutaneous MTP1 fusion is a simple and quick procedure which can achieve functional results comparable to those obtained with open MTP1 fusion with more than 90% of patients satisfied [Womack [7], Goucher [13], Coughlin [19], Flavin [20], Brodsky [21], Yee [22].

In open procedures for MTP1 fusion, the method of bone preparing requires a large approach with a risk of postoperative prolonged pain and swelling or wound healing difficulties Kelikian [6], Womack [7]. One of the benefits of the percutaneous MTP1 fusion is the decreased morbidity (few pain and few scar problems) with the possibility of performing this procedure in outpatients with immediate full weight bearing.

Bone preparation is a crucial step of this procedure and requires an experience in percutaneous forefoot surgery. In this technique, bone cuts are flat and any mistake on the preparation will have an impact on the positioning of the arthrodesis. Bone resection with the burr must be controlled to avoid any bone loss or assymmetrical resection that would affect primary stability, bone contact of the arthrodesis and lead to excessive shortening of the first ray. A cup-and-cone configuration of bone preparation is more sound than flat bone cuts either for biomechanical reasons and for the arthrodesis positioning that is simpler without first ray shortening [Curtis [10], Goucher [13]. However this method of preparing the fusion site is difficult to perform with a percutaneous approach.

Arthrodesis positioning is perhaps the most critical of all the technical considerations. It is not only a problem of alignment of the great toe in terms of valgus/varus, dorsal flexion/plantar flexion or medial rotation/lateral rotation but also a question of metatarsus varus, metatarsal length, hindfoot positioning (valgus flatfoot, pes cavus), forefoot symptoms (metatarsalgias, lesser toes deformities) and shoe wear habits (flat shoes or high heel shoes) [Conti [3], Harper [4], Alexander [5], Kelikian [6], Womack [7]. Arthrodesis positioning is easy to perform with the percutaneous technique and can be assessed at any time either clinically and under fluoroscopic control. A metallic support materializing the floor plane is very useful to have a good positioning of the great toe Kelikian [6], Womack [7].

The fixation with cannulated compression crossed screws is a very simple technique but is not biomechanically the most stable technique. It is therefore important to assess with accuracy the position of the screws to provide a good compression Womack [7].

## CONCLUSION

Percutaneous MTP1 fusion is a simple procedure providing comparable results to fusions performed with open techniques. Postoperative cares are simplified with an immediate full weight bearing on rigid flat shoes and quick return to normal walking. Bone preparation is an important step and requires an experience in percutaneous forefoot surgery. Arthrodesis positioning and fixation with this percutaneous procedure are simple with possibility of clinical and radiological control. The indications for percutaneous MTP1 fusion are very large and only severe bone loss or osteoporosis represent the limits for this technique.
